# Blockade of insulin receptor signaling in the medullary cardiovascular centers impairs open‐loop arterial baroreflex function via attenuated neural arc in healthy male rats

**DOI:** 10.1096/fj.202403097R

**Published:** 2025-02-27

**Authors:** Amane Hori, Toru Kawada, Norio Hotta, Ayumi Fukazawa, Juan A. Estrada, Han‐Kyul Kim, Gary A. Iwamoto, Scott A. Smith, Wanpen Vongpatanasin, Masaki Mizuno

**Affiliations:** ^1^ Department of Applied Clinical Research University of Texas Southwestern Medical Center Dallas Texas USA; ^2^ College of Life and Health Sciences Chubu University Kasugai Japan; ^3^ Japan Society for the Promotion of Science Tokyo Japan; ^4^ Department of Cardiovascular Dynamics National Cerebral and Cardiovascular Center Suita Japan; ^5^ Department of Internal Medicine University of Texas Southwestern Medical Center Dallas Texas USA; ^6^ Department of Surgery University of Texas Southwestern Medical Center Dallas Texas USA

**Keywords:** blood pressure regulation, central nervous system, insulin, insulin receptor signaling, open‐loop baroreflex analysis

## Abstract

Evidence suggests that brain insulin availability acutely modulates arterial baroreflex function. However, little is known about the impact of blocking brain insulin receptor (IR) signaling on arterial baroreflex. We hypothesized that blockade of IR signaling in the brain acutely impairs arterial baroreflex function. Our hypothesis was tested using baroreflex open‐loop analysis to evaluate the two subsystems of the arterial baroreflex: the carotid sinus pressure (CSP)–sympathetic nerve activity (SNA) relationship (the neural arc) and the SNA–arterial pressure (AP) relationship (the peripheral arc). In anesthetized healthy male rats, the bilateral carotid sinus baroreceptor regions were surgically isolated from the systemic circulation, and then CSP was changed stepwise from 60 to 180 mmHg before and over 120 min after lateral intracerebroventricular (ICV) administration of either artificial cerebrospinal fluid (control solution) or IR antagonist GSK1838705. ICV injection of GSK1838705 significantly decreased renal SNA (RSNA), AP, and heart rate during stepwise CSP input over a period of 120 min after administration (*p* < .05). The maximum gain of the neural arc was significantly reduced 120 min after ICV injection of GSK1838705 (*p* = .002). Furthermore, GSK1838705 significantly attenuated the operating‐point RSNA (*p* = .025) and AP (*p* < .001) as estimated by the baroreflex equilibrium diagram. Moreover, 120‐min baroreflex stimulation via stepwise CSP input significantly increased c‐Fos expression in IR‐positive neurons in medullary cardiovascular centers (*p* < .001). Our findings suggest that IR signaling in the brain can modulate AP regulation via alteration of the neural arc of the arterial baroreflex.

## Graphical abstract

The present study demonstrates that intracerebroventricular injection of an insulin receptor (IR) antagonist acutely impairs the neural arc, but not the peripheral arc, component of arterial baroreflex function under open‐loop conditions. Furthermore, the operating points of renal sympathetic nerve activity and arterial pressure are reduced by IR blockade in the brain. These results suggest that IR signaling in the medullary cardiovascular centers plays a crucial role in regulating arterial pressure via the neural arc function of the arterial baroreflex.

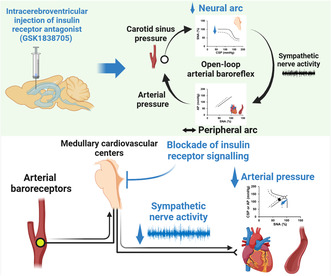

**Abbreviations**
aCSF: artificial cerebrospinal fluid
ANOVA: analysis of variance
AP: arterial pressure
BSA: bovine serum albumin
CSF: cerebrospinal fluid
CSP: carotid sinus pressure
CVLM: caudal ventrolateral medulla
DAPI: 4′,6‐diamidino‐2‐phenylindole
DM: diabetes mellitus
DMSO: dimethyl sulfoxide
ECG: electrocardiogram
HR: heart rate
ICV: intracerebroventricular
IR: insulin receptor
NGS: normal goat serum
NTS: nucleus tractus solitarius
PBS: phosphate‐buffered saline
RSNA: renal sympathetic nerve activity
RVLM: rostral ventrolateral medulla
SNA: sympathetic nerve activity
Vsp: spinal trigeminal nucleus

## 1 INTRODUCTION

Insulin in the periphery is well known to play an important role in the regulation of glucose and lipid metabolism.^1^ Increasing evidence demonstrates that insulin also modulates neural activity in the central nervous system. For example, exogenous insulin delivery into the brain activates the sympathetic nervous system.^2^, ^3^, ^4^, ^5^ However, it remains to be fully elucidated how brain insulin modifies autonomic cardiovascular regulation.

The arterial baroreflex is a negative feedback system that modulates autonomic nerve activity to maintain arterial pressure (AP) at rest and during physical activity. Arterial baroreflex dysfunction is known to induce AP lability,^6^ making high AP variability a risk factor for the development of cardiovascular disease and mortality.^7^ Evidence suggests that brain insulin availability may acutely modulate arterial baroreflex function. For example, Pricher et al.^8^ reported that lateral intracerebroventricular (ICV) injection of insulin potentiates arterial baroreflex function. Additionally, Daubert et al.^9^ found impaired arterial baroreflex function and decreased cerebrospinal fluid (CSF) insulin levels in pregnant animals. Furthermore, a follow‐up study demonstrated that ICV infusion of insulin improves the impaired arterial baroreflex function in pregnant rats.^10^ Moreover, it is well known that the action of insulin depends not only on the amount of insulin available, but also on the downstream signaling pathway at the insulin receptor (IR).^11^, ^12^ In support of this concept, impaired brain IR signaling, observed in diabetes mellitus (DM),^13^, ^14^ has been implicated in the generation of AP lability due to baroreflex impairment.^15^ However, to date, it remains to be elucidated whether changes in downstream IR signaling (achieved experimentally by blocking endogenous IR directly) alter baroreflex function.

The arterial baroreflex is divided into the following two principal subsystems: the neural arc, which determines sympathetic nerve activity (SNA) in response to a baroreceptor pressure input, and the peripheral arc, which determines AP in response to SNA.^16^ Under normal physiological conditions, the arterial baroreflex operates as a closed‐loop negative feedback system. However, the neural and peripheral arcs usually cannot be evaluated under the baroreflex closed‐loop conditions since AP cannot be separated into the input and output pressures. Furthermore, for the same reason, the baroreflex closed‐loop conditions usually do not allow evaluation of baroreflex control of AP (i.e., the total reflex arc that determines the AP in response to the baroreceptor pressure input). With these experimental considerations in mind, to precisely assess arterial baroreflex function, a baroreflex open‐loop analysis must be performed. This can be established by surgically isolating the carotid sinus baroreceptor area from the systemic circulation.^16^

The purpose of this study was therefore to investigate the effects of an ICV injection of IR antagonist on arterial baroreflex function under open‐loop conditions in healthy male rats. We hypothesized that the blockade of IR signaling in the brain acutely impairs arterial baroreflex function via alterations in the neural arc, but not the peripheral arc, independent of circulating insulin and glucose levels. It is well known in the sympathetic baroreflex that carotid sinus baroreceptors project via sensory afferents to the nucleus tractus solitarius (NTS) followed by the caudal ventrolateral medulla (CVLM) and then the rostral ventrolateral medulla (RVLM) in sympathetic baroreflex function.^17^, ^18^ Moreover, insulin receptors are known to be expressed in numerous areas throughout the brainstem involved in regulating the cardiovascular system.^19^ Thus, we further investigated 1) whether IR‐positive neurons co‐localize with c‐Fos (a marker of neuronal activation)‐positive neurons activated by baroreflex stimulation in the NTS, the CVLM, and the RVLM by using immunofluorescence staining, and 2) whether blockade of IR signaling potentiates AP lability against hypertensive/hypotensive stress using baroreflex equilibrium diagram simulation.

## 2 METHODS

### 2.1 Ethical approval

This study was performed in accordance with the U.S. Department of Health and Human Services NIH *Guide for the Care and Use of Laboratory Animals*. The Institutional Animal Care and Use Committee of the University of Texas Southwestern Medical Center approved this study (no. 2019–102 849), and all experimental procedures were conducted following approved institutional guidelines and regulations.

### 2.2 Animals

Thirty‐five healthy male Sprague–Dawley rats (12–16 weeks, body weight: 384 ± 26 g) (Inotiv, West Lafayette, IN, USA) were used for the fluorescence immunohistochemistry and the baroreflex open‐loop in vivo experiment. The animals had free access to food and clean water and were kept one to four per cage under a 12 h light–dark cycle in an air‐conditioned room (22–24°C) until required for terminal experiments.

### 2.3 Fluorescence immunohistochemistry

In six anesthetized rats, the bilateral carotid sinus baroreceptor regions were surgically isolated from the systemic circulation. The surgical and anesthesia procedures for the preparation of the baroreflex stimulation under the open‐loop conditions were described in the “*Open‐loop baroreflex in vivo experiment*” section below. We confirmed the adequacy of anesthesia by lack of a withdrawal response to tail pinch. In the baroreflex stimulation treated group (*n* = 3), following the 60‐min post‐surgery recovery period, repetitive baroreflex stimulation was performed. By the procedure described below, carotid sinus pressure (CSP) was first decreased to 60 mm Hg for 4 min and then increased in a stepwise manner up to 180 mmHg in increments of 20 mmHg every minute. Peak c‐Fos expression is known to occur approximately 60–120 min following the neural activation.^20^, ^21^ Thus, the CSP input cycle was repeated for 120 min, followed by a 60‐min resting period under the closed‐loop conditions where CSP was matched to AP. In contrast, the control group (*n* = 3) was under the closed‐loop conditions for the same period (i.e., 3 h) after the 60‐min post‐surgery recovery period. Immediately after the 60‐min resting period, transcardial perfusion with physiological saline was performed for 15 min followed by 15‐min 4% paraformaldehyde for tissue fixation. Thus, the tissue fixation was achieved within approximately 90 min after the baroreflex stimulation period. The brain tissue was harvested and post‐fixed overnight in 4% paraformaldehyde followed by dehydrated sequentially in 10% and 20% sucrose.

The present immunofluorescence staining was adopted from our previous study.^22^ Briefly, after submerging in an optimal cutting temperature medium and freezing on dry ice, the brainstem tissue was sectioned at 35 μm using a cryostat (2800 Frigocut, Reichert Jung/Leica, Deer Park, IL, USA). The sections were washed in phosphate‐buffered saline (PBS). After blocking with an antibody buffer solution (PBS, 0.5% Triton‐X100, 5% normal goat serum [NGS], and 10 mg/mL bovine serum albumin [BSA]) for 60 min, co‐immunostaining for IR and c‐Fos was performed by incubation with the primary antibodies (mouse anti‐IRβ, 1:100, cat. no. sc‐57 342, RRID#AB_784102, Santa Cruz Biotechnology, Dallas, TX, USA; rabbit anti‐c‐Fos, 1:1000, cat. no. 2250, RRID#AB_2247211, Cell Signaling Technology, Danvers, MA, USA) overnight at room temperature. The sections were rinsed in PBS with 5% NGS and 10 mg/mL BSA and then incubated with fluorescence‐conjugated secondary antibodies (goat anti‐mouse Cy3, 1:500, cat. no. 115–165‐146, RRID#AB_2338690, Jackson ImmunoResearch Laboratories, West Grove, PA, USA; goat anti‐rabbit Alexa Fluor Plus 488, 1:500, cat. no. A32731, RRID#AB_2633280, Thermo Fisher Scientific, Waltham, MA, USA) for 60 min at room temperature. We previously used the same primary and secondary antibody combinations to perform the triple labeling of IR, c‐Fos, and DAPI in the NTS.^22^ Then, sections were rinsed with PBS with 5% NGS and 10 mg/mL BSA and pure water, and then fixed onto charged slides. The mounting medium with 4′,6‐diamidino‐2‐phenylindole (DAPI) (Mounting Medium With DAPI, cat. no. ab104139, Abcam, Cambridge, MA, USA) was applied to charged slides before coverslipping with micro cover glass. We performed negative control experiments to ensure the specificity of the antibodies.

A fluorescence microscope system (Axio Imager A2, Zeiss, Oberkochen, Germany) was used for observing and obtaining the fluorescence images as described previously.^22^, ^23^ To evaluate whether c‐Fos‐positive neurons activated by baroreflex stimulation were specifically observed in medullary cardiovascular centers (the NTS, CVLM, and RVLM), we additionally investigated the degree of IR, c‐Fos, and DAPI overlap in the spinal trigeminal nucleus (Vsp) which is believed not to be activated by arterial baroreflex. The NTS, CVLM, RVLM, and Vsp were, respectively, determined as the regions from about −15.4 to −13.6 mm, −13.4 to −13.0 mm, −12.4 to −12.0 mm, and −15.4 to −12.0 mm to the bregma based on the rat brain atlas and previous studies.^22^, ^23^, ^24^ For quantitative analysis, the sections of the NTS (10 sections each rat), CVLM (3 sections each rat), and RVLM (3 sections each rat) were serially selected at approximately 2‐mm intervals from each rat. As for the Vsp, we collected three sections from −15.4 to −13.6 mm to the bregma region and one section each from −12.4 to −12.0 mm to the bregma and from −13.4 to −13.0 mm to the bregma regions (i.e., total 5 sections each rat). A series of grayscale photomicrographs at 10× magnification were acquired, and then we performed triple overlap analysis using image analysis software (FIJI; National Institute of Health, Maryland, USA) as we did previously.^22^ Briefly, the image background was subtracted within the region of interest. Subsequently, individualized thresholds (Triangle threshold, c‐Fos; Otsu threshold, IR and DAPI), and then particle size filters were applied for each binary image. Finally, IR^+^, c‐Fos^+^, and DAPI^+^ areas were each calculated, as well as calculating the overlap between IR^+^/c‐Fos^+^/DAPI^+^ areas.

### 2.4 Open‐loop baroreflex in vivo experiment

#### 2.4.1 General surgical experimental procedures

Rats were initially anesthetized with an intraperitoneal injection of a cocktail consisting of urethane (800 mg/kg) and α‐chloralose (65 mg/kg), and then mechanically ventilated with 100% oxygen gas after intubation. To maintain the depth of anesthesia, a 7‐fold dilution of the above anesthetic cocktail was continuously infused via the right femoral vein at a rate of 3–5 mL/h/kg. The depth of anesthesia was evaluated and monitored by tail pinch until all protocol was completed. Body temperature was maintained at approximately 36.5–37°C using a heating pad and a heat lamp. A catheter was inserted into the right femoral artery and connected to a pressure transducer (MLT0670, ADInstruments, Sydney, Australia) to measure AP. Electrocardiogram (ECG) recordings were obtained using needle electrodes. For recording renal SNA (RSNA), a branch of the isolated left renal nerve was attached to the electrodes (Platinum, AM Systems, Sequim, WA, USA) and covered with silicone glue (Kwik‐Sil, World Precision Instruments, Sarasota, FL, USA) for insulation and fixation. To validate that most of the RSNA recording was obtained from postganglionic renal sympathetic fibers, hexamethonium bromide (60 mg/kg) was administered intravenously after all experiments were completed. Additionally, RSNA background noise was measured over a 30‐min period after animals were euthanized by intravenous injection of saturated potassium chloride (4 M, 2 mL/kg) under anesthesia. For ICV injection, animals were placed on a stereotaxic head unit (David Kopf Instruments, Tujunga, CA, USA). A small hole was first drilled into the skull, and then a 35‐gauge needle (WPI, Sarasota, FL, USA) was implanted in the right lateral ventricle using coordinates of 0.9 mm caudal, 1.8 mm lateral, and 3.6 mm ventral to the bregma.^24^

To create an open‐loop baroreflex condition, the bilateral carotid sinus baroreceptor regions were isolated from the systemic circulation using previously described methods.^25^, ^26^, ^27^ In brief, the external carotid artery was ligated close to the carotid bifurcation. Subsequently, the internal carotid artery was embolized with 0.8‐mm diameter steel balls (SBM‐SUJ‐0.8, Tsubaki Nakashima, Nara, Japan), and the common carotid artery was catheterized. Physiological saline was then used to fill the isolated carotid sinuses and catheters. Using a servo‐pump system (ET‐126, Labworks, Costa Mesa, CA, USA), CSP was controlled via catheters inserted into the common carotid arteries. To minimize cardiovascular reflexes from the cardiopulmonary region and aortic arch, bilateral vagal and aortic depressor nerves were sectioned at the neck.

Following the 60 min post‐surgery recovery period, CSP was first decreased to 60 mmHg for 4 min, and then increased stepwise from 60 to 180 mmHg in increments of 20 mmHg every minute according to previous studies.^25^, ^26^, ^28^ The stepwise CSP input cycle was repeated throughout the protocol.^26^, ^29^ Before evaluating AP, RSNA, and heart rate (HR) responses to the stepwise CSP input, blood was collected from the tail vein for assessing blood glucose and plasma insulin levels in some animals. Blood was obtained from the tail vein to reduce the potential negative impact that collecting blood from the jugular vein could have on arterial baroreflex function. Then, ICV injection of either 1 mM GSK1838705 (Sigma‐Aldrich, St. Louis, MO, USA) (1 μL/1 min), an IR antagonist, or artificial cerebrospinal fluid (aCSF, Harvard Apparatus, Holliston, MA, USA) (1 μL/1 min) as a control solution was commenced by using a microsyringe (Hamilton syringe; VWR, Missouri City, TX, USA) mounted on a micropump (UMP3, WPI). The final concentration of GSK1838705 was 1 mM, diluted in a solution of aCSF containing 50% dimethyl sulfoxide (DMSO, Sigma‐Aldrich) (1:1 aCSF and DMSO). Since it has been reported that RSNA increases approximately 120 min after the ICV injection of insulin,^4^ we anticipated that it would take several hours before significant effects of the ICV injection of the IR antagonist on arterial baroreflex function would manifest. Thus, we recorded AP, RSNA, and HR responses to the CSP input at 30, 60, 90, and 120 min after ICV injection. As a set of corollary experiments, we tested whether the 1:1 aCSF and DMSO solution changes the open‐loop baroreflex function. Again, venous blood was collected from the tail vein after recording the 120‐min data after ICV injection in some animals.

The collected blood sample was assessed for blood glucose by using a handheld glucose meter (FreeStyle Precision Neo; Abbott, Chicago, IL, USA). Plasma insulin was assayed using an enzyme‐linked immunosorbent assay kit (Ultra‐Sensitive Rat Insulin ELISA Kit, catalog no. 90060, Crystal Chem, Elk Grove Village, IL, USA).

#### 2.4.2 Data analysis

RSNA, AP, CSP, and ECG signals were amplified, filtered, and continuously recorded on a computer at a 1 kHz sampling rate via the analog‐to‐digital converter (PowerLab 8/30, ADInstruments). Data analysis was performed by LabChart 8 application software (ADInstruments). HR was calculated from the ECG recording. For analyzing RSNA, the preamplified nerve signal was band‐pass filtered at 100–1000 Hz (Neuro Amp EX; ADInstruments) and then low‐pass filtered with a cutoff frequency of 30 Hz using the LabChart 8 application software. Full‐wave rectified signals of RSNA were subsequently used for quantification. AP, RSNA, and HR data were averaged for the last 10 s at each CSP level. To quantify the RSNA response to the stepwise CSP input, the RSNA value of the last 10 s at a CSP of 60 mmHg before ICV injection was designated as 100% baseline, and the stimulation‐induced changes in RSNA were expressed as a percentage of this baseline.

The input–output relationships between CSP and RSNA (the neural arc), between CSP and AP (the total reflex arc), and between CSP and HR (HR control) were described by fitting the following four‐parameter logistic function to the data points as follows.^16^, ^30^

$$y=\frac{P_{1}}{1+\exp\left[P_{2}\left(\mathrm{CSP}-P_{3}\right)\right]}+P_{4}$$

where *y* represents the output value (RSNA, AP, or HR), *P*_1_ is the response range, *P*_2_ is the slope coefficient, *P*_3_ is the midpoint pressure on the CSP axis, and *P*_4_ is the minimum value of output. The maximum gain of the logistic function was calculated from −*P*_1_ *P*_2_/4.

The input–output relationship between SNA and AP (the peripheral arc) was described by linear regression as follows^16^:

$$\mathrm{AP}=b_{0}+b_{1}\times \text{RSNA}$$

where *b*_0_ and *b*_1_ represent the intercept and slope of the regression line, respectively.

To estimate the operating point, using RSNA as the common abscissa and CSP or AP as the ordinate, the baroreflex equilibrium diagram was drawn by plotting data from the neural and peripheral arcs averaged for the last 10 s at each CSP level.^16^, ^31^ The operating‐point RSNA and AP were determined from the intersection of the fitted neural and peripheral arcs on the baroreflex equilibrium diagram.

### 2.5 Baroreflex equilibrium diagram simulation under hypertensive and hypotensive stresses

Following previously described methods,^32^ the imposition of external disturbance (hypertensive stress [+5, +10, +15, and +20 mmHg] and hypotensive stress [−5, −10, −15, and −20 mmHg]) was simulated using the data from the open‐loop baroreflex in vivo experiment. The peripheral arc in the baroreflex equilibrium diagram was shifted upward (+5, +10, +15, and +20 mmHg) to mimic hypertensive stress and downward (−5, −10, −15, and −20 mmHg) to simulate hypotensive stress. Then, the operating‐point AP was estimated by the intersection of the neural arc and peripheral arc whose intercept was changed under hypertensive/hypotensive stress before and after ICV injection of control or IR antagonist solution. We determined the operating‐point AP rise and fall (Δoperating‐point AP) by calculating the difference in operating‐point AP from before to after hypertensive or hypotensive stress for evaluating AP lability against the imposition of external disturbance.

### 2.6 Statistical analysis

The Shapiro–Wilk test was first performed to confirm data normality. In the histological data, we performed an unpaired t‐test or the Mann–Whitney U‐test, appropriately. Additionally, for evaluating the distribution of IR, c‐Fos, and DAPI expression, as well as the overlap area between IR, c‐Fos, and DAPI expression in the NTS, a two‐way repeated measures analysis of variance (ANOVA) was performed (group‐by‐coordinate). If an interaction and/or a main effect was significant, Bonferroni's multiple comparison test was conducted. In the physiological data, RSNA, AP, and HR at each CSP level before and 30, 60, 90, and 120 min after ICV injection of each solution were analyzed using a two‐way repeated measures ANOVA (time‐by‐CSP). If an interaction and/or a main effect was significant, Bonferroni's multiple comparison test was performed. For the analysis of parameters of static characteristics of the neural arc, peripheral arc, total reflex arc, heart HR control, operating‐point AP and RSNA, blood glucose, and plasma insulin before and 120 min after ICV injection control and GSK1838705 solutions, a two‐way repeated measures ANOVA was performed (solution‐by‐time). If an interaction and/or a main effect was significant, we performed Bonferroni's multiple comparison test. A one‐way repeated measures ANOVA or Friedman test was utilized for the parameters of static characteristics before and 30, 60, 90, and 120 min after ICV injection of 1:1 aCSF and DMSO solution. If a one‐way repeated measures ANOVA or Friedman test detected significance, Bonferroni's or Dunn's multiple comparisons test was performed. A paired t‐test or Wilcoxon signed‐rank test was used for analyzing blood glucose and plasma insulin before and 120 min after ICV injection of the 1:1 aCSF and DMSO solution as appropriate. In the simulation data, a two‐way repeated measures ANOVA was performed (time‐by‐pressure). If an interaction and/or a main effect was significant, Bonferroni's multiple comparison test was used. Moreover, we calculated the changes in ΔAP from before to 120 min after ICV injection of control or IR antagonist, and a two‐way repeated measures ANOVA was performed (solution‐by‐pressure). If an interaction and/or a main effect was significant, we performed Bonferroni's multiple comparison test.

All statistical analyses were computed using statistical software (SPSS Statistics 28, IBM, Armonk, NY, USA). Statistical significance was defined as *p* < .05. Data are presented as the mean ± SD.

## 3 RESULTS

### 3.1 Fluorescence immunohistochemistry

**FIGURE 1:**
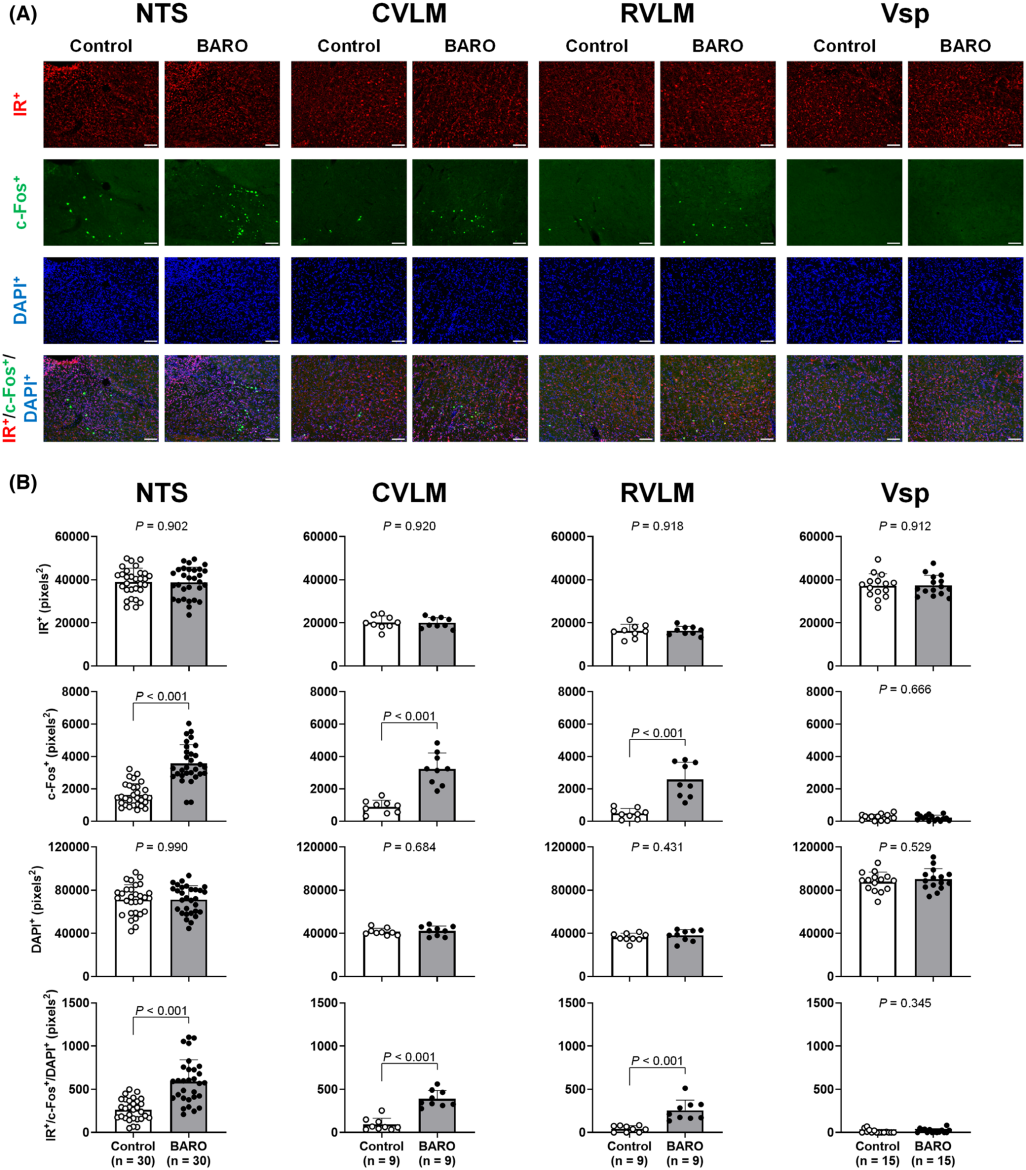
The area of insulin receptor (IR), c‐Fos, and 4′,6‐diamidino‐2‐phenylindole (DAPI) expression, and the overlap area between IR, c‐Fos, and DAPI expression in the nucleus tractus solitarius (NTS), caudal ventrolateral medulla (CVLM), rostral ventrolateral medulla (RVLM), and spinal trigeminal nucleus (Vsp) in control and baroreflex stimulation (BARO) treated rats. Representative image of the expression of IR, c‐Fos, and DAPI and co‐localization with IR, c‐Fos, and DAPI in the NTS, CVLM, RVLM, and Vsp (A). The sections of the NTS (10 sections each rat), CVLM (3 sections each rat), RVLM (3 sections each rat), and Vsp (5 sections each rat) were collected from three control and three BARO‐treated rats. In the BARO‐treated group, carotid sinus pressure (CSP) was changed stepwise under open‐loop conditions, while control rats were rested under closed‐loop conditions (CSP was matched to arterial pressure by using a servo‐pump system). The magnification is ×10. The white scale bar is 100 μm. The data were analyzed by an unpaired t‐test or the Mann–Whitney *U*‐test (B). Data are shown as mean ± SD.

**FIGURE 2:**
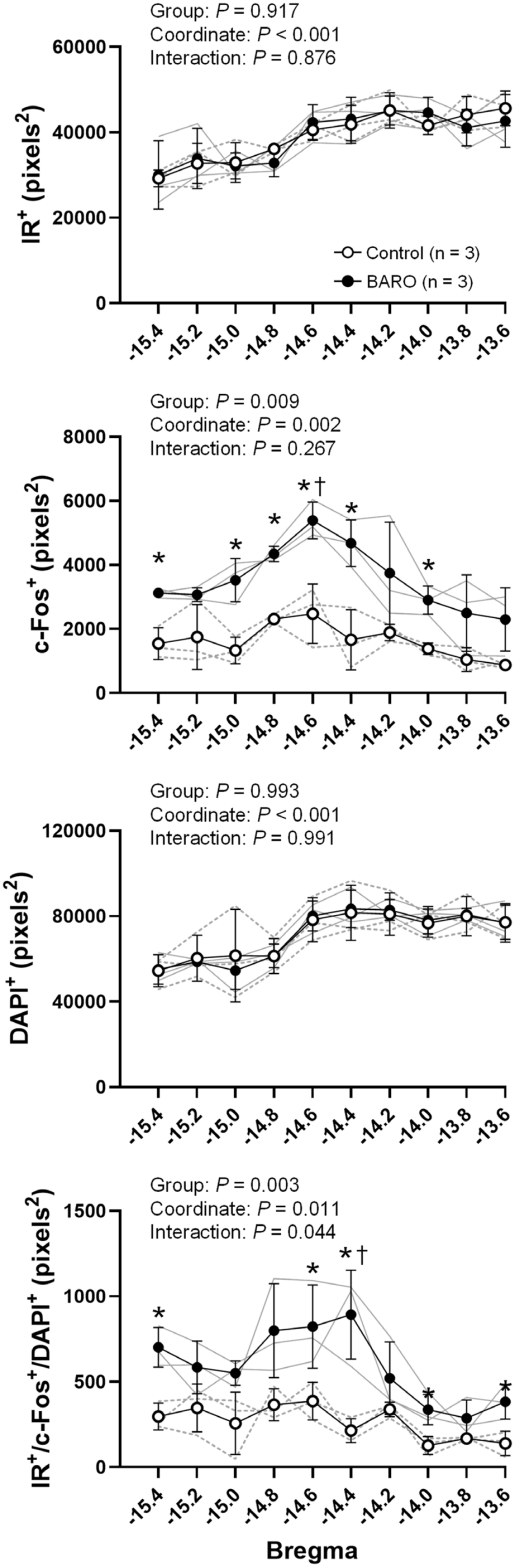
Distribution of insulin receptor (IR), c‐Fos, and 4′,6‐diamidino‐2‐phenylindole (DAPI) expression, and the overlap area between IR, c‐Fos, and DAPI expression at each bregma level of the nucleus tractus solitarius (NTS) in control and baroreflex stimulation (BARO) treated rats. In the BARO treated group, carotid sinus pressure (CSP) was changed stepwise under open‐loop conditions while control rats were rested under closed‐loop conditions (CSP was matched to arterial pressure by using a servo‐pump system). Sections of the NTS (10 sections each rat) were serially collected from three control and three BARO treated rats. The data were analyzed by a two‐way repeated measures ANOVA (group‐by‐coordinate) followed by Bonferroni's multiple comparison test. *p < .05 vs. control group. ^†^ *p* < .05 vs. −13.6 mm to the bregma. The gray dashed and solid lines show individual data from the control and the BARO treated groups, respectively. Data are shown as the mean ± SD.

Figure 1A shows the individual representative images and co‐localization of IR, c‐Fos, and DAPI in neurons in the NTS, CVLM, RVLM, and Vsp. The representative images were pseudo‐colored from grayscale for illustration. The area of DAPI‐ and IR‐positive neurons in the NTS, CVLM, and RVLM did not differ significantly between baroreflex stimulation and control groups (Figure 1B). The 120‐min repetitive baroreflex stimulation significantly increased c‐Fos protein in the NTS, CVLM, and RVLM, but not in the Vsp (Figure 1B). Furthermore, the overlap area between IR^+^/c‐Fos^+^/DAPI^+^ in the NTS, CVLM, and RVLM was significantly increased by the baroreflex stimulation (Figure 1B). The IR, c‐Fos, and DAPI overlap area in the Vsp, as the negative control area, was not significantly different among trials (Figure 1B). Figure 2 shows the distribution of IR^+^, c‐Fos^+^, and DAPI^+^, as well as the overlap between IR^+^, c‐Fos^+^, and DAPI^+^ in the NTS. In DAPI‐ and IR‐positive neurons, there were no significant differences between the groups at any coordinates in the NTS. Areas of c‐Fos‐positive neurons and the triple overlap in the baroreflex stimulation group were significantly greater than those in the control group at several coordinates in the NTS.

### 3.2 Open‐loop baroreflex in vivo experiment

As compared to the baseline, blood glucose and plasma insulin were not significantly changed 120 min after ICV injection of control (aCSF) and GSK1838705 solutions (blood glucose; before: 132 ± 30 mg/dL, 120 min: 132 ± 21 mg/dL in control solution [*n* = 5] vs. before: 122 ± 9 mg/dL, 120 min: 125 ± 14 mg/dL in GSK1838705 solution [*n* = 5]; solution effect: *p* = .436; time effect: *p* = .775; interaction: *p* = .775; plasma insulin; before: 2.0 ± 0.8 ng/mL, 120 min: 1.2 ± 0.3 ng/mL in control solution [*n* = 5] vs. before: 3.3 ± 1.6 ng/mL, 120 min: 2.6 ± 2.5 ng/mL in GSK1838705 solution [*n* = 3]; solution effect: *p* = .168; time effect: *p* = .086; interaction: *p* = .903). ICV injection of aCSF containing 50% DMSO (GSK1838705 vehicle solution) also did not significantly change the blood glucose (before: 119 ± 22 mg/dL vs. after: 125 ± 30 mg/dL, *p* = .22 [*n* = 5]) and plasma insulin (before: 1.8 ± 0.7 ng/mL vs. after: 1.1 ± 0.3 ng/mL, *p* = .067 [*n* = 5]).

**FIGURE 3:**
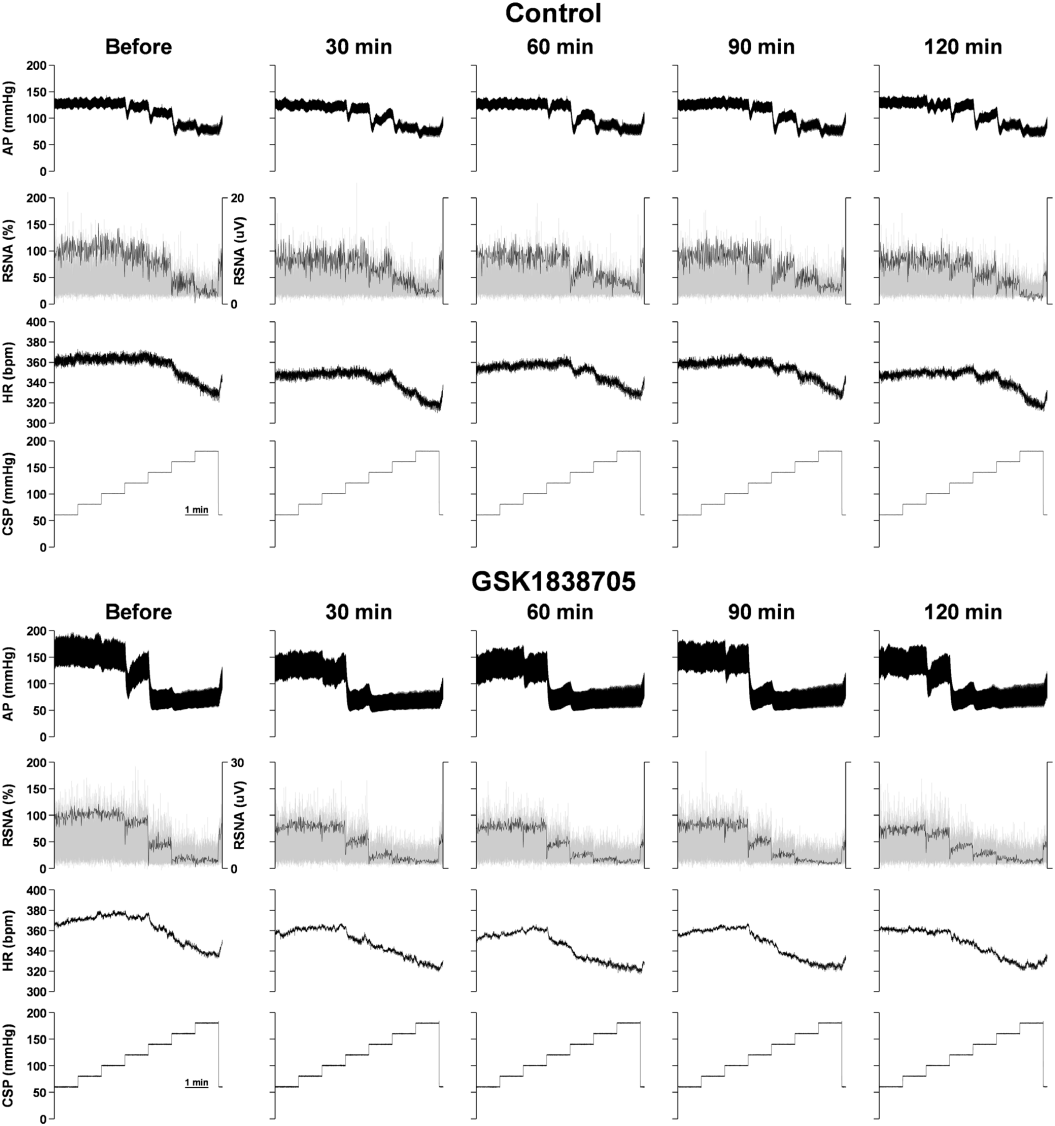
Representative recordings of arterial pressure (AP), renal sympathetic nerve activity (RSNA), and heart rate (HR) responses to stepwise carotid sinus pressure (CSP) input before and 30, 60, 90, and 120 min after intracerebroventricular injection of artificial cerebrospinal fluid (control) and insulin receptor antagonist (GSK1838705). Gray and black lines in the RSNA show rectified raw data and normalized data, respectively.

**FIGURE 4:**
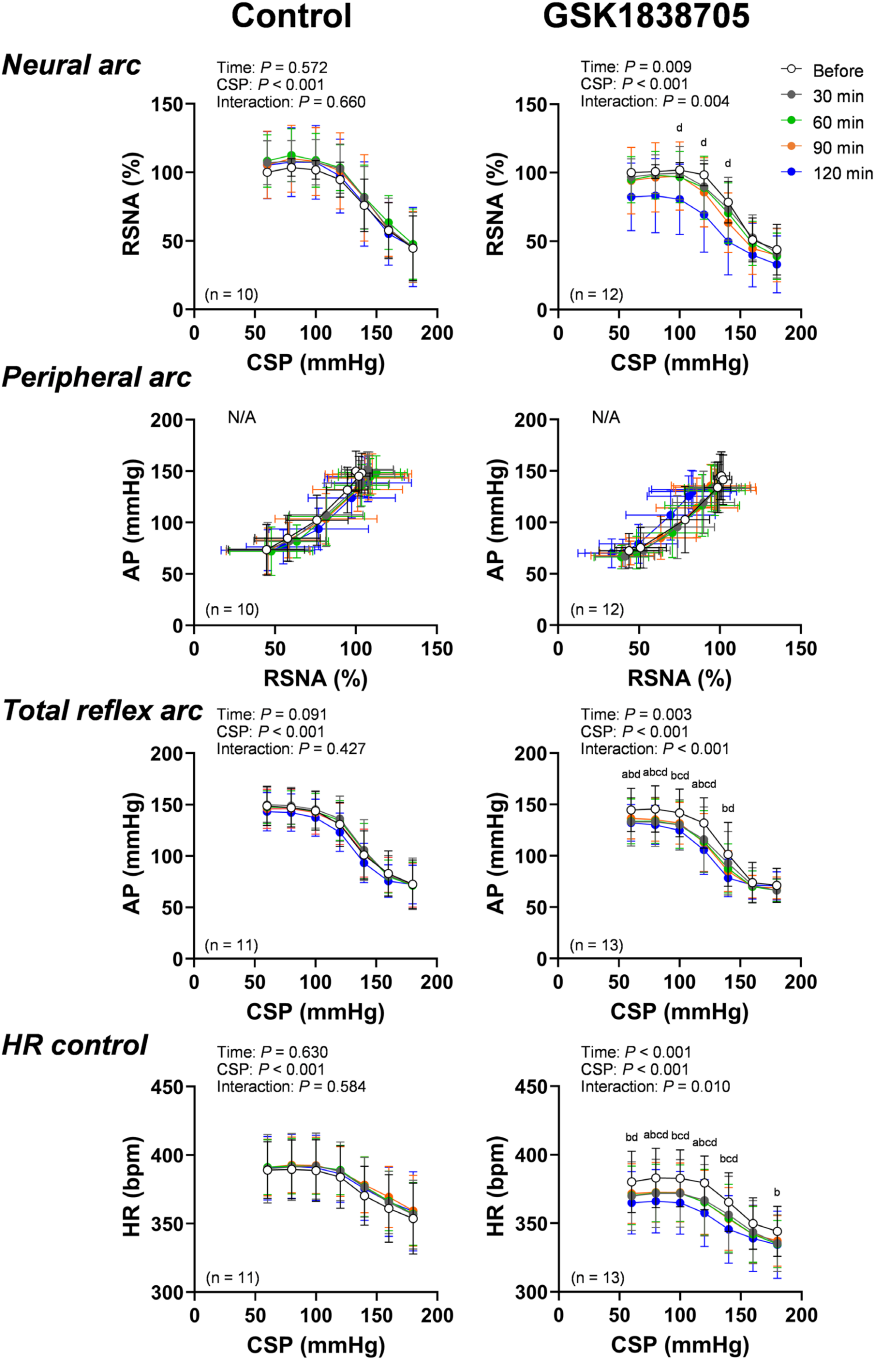
Open‐loop static characteristics of the neural arc, peripheral arc, total reflex arc, and heart rate (HR) control before and 30, 60, 90, and 120 min after intracerebroventricular injection of artificial cerebrospinal fluid (control) and insulin receptor antagonist (GSK1838705). CSP, carotid sinus pressure; RSNA, renal sympathetic nerve activity; AP, arterial pressure. The data were analyzed by a two‐way repeated measures ANOVA (time‐by‐CSP) followed by Bonferroni's multiple comparison test. a, b, c, and d show significant differences between before and 30, 60, 90, or 120 min, respectively (*p* < .05). Since the x‐axis in the peripheral arc was RSNA, which varied between animals, a two‐way repeated measures ANOVA was not performed for the peripheral arc. Data are shown as the mean ± SD.

Figure 3 shows representative recordings of AP, RSNA, and HR responses to stepwise CSP input before and 30, 60, 90, and 120 min after ICV injection of control and GSK1838705 solutions. We successfully measured AP and HR in 24 rats and RSNA in 22 rats. A stepwise increase in CSP decreased RSNA, AP, and HR. Analysis of trial‐averaged static characteristics of the baroreflex demonstrated that the interactions (time‐by‐CSP) of the neural arc, total reflex arc, and HR control were significant in GSK1838705 but not in the control trial (Figure 4). Moreover, ICV injection of GSK1838705 but not that of control significantly decreased AP, RSNA, and HR at each CSP level (Figure 4). Since the effects of GSK1838705 were more pronounced at 120 min after the ICV injection, we used the values before and 120 min after ICV injection for comparisons of each parameter of the neural arc, peripheral arc, total reflex arc, and HR control.

Table 1 shows the parameters of static characteristics of the neural arc, peripheral arc, total reflex arc, and HR control before and 120 min after ICV injection of control and GSK1838705 solutions. In the neural arc, while the response range (*P*_1_), slope coefficient (*P*_2_), midpoint pressure (*P*_3_), and minimum value (*P*_4_) were not significantly changed by ICV injection of the GSK1838705 solution, the maximum gain (*G*_max_) was significantly decreased after ICV injection of GSK1838705. In the peripheral arc, intercept (*b*_0_) and slope (*b*_1_) were not significantly altered by ICV injection of either test solution. In the total reflex arc, although the response range (*P*_1_) and midpoint pressure (*P*_3_) were significantly decreased regardless of the test solutions, the significant interaction between solution (control vs. GSK1838705) and time (before vs. 120 min) was not observed in any parameters of the total reflex arc. In HR control, ICV injection of both solutions significantly decreased the response range (*P*_1_) without significantly changing the slope coefficient (*P*_2_) and midpoint pressure (*P*_3_). The minimum value (*P*_4_) after ICV injection of GSK1838705 was significantly lower than that after ICV injection of the control solution. GSK1838705, but not the control, significantly decreased the maximum gain (*G*_max_).

**TABLE 1:** Parameters of static characteristics of the neural arc, peripheral arc, total reflex arc, and heart rate (HR) control before and 120 min after intracerebroventricular injection of artificial cerebrospinal fluid (control) and insulin receptor antagonist (GSK1838705).

		Before	120 min	Solution	Time	Interaction
Neural arc						
*P*_1_, Response range (%)	Control (*n* = 10)	62.0 ± 26.8	64.4 ± 25.3	*p* = .382	*p* = .501	*p* = .155
	GSK1838705 (*n* = 12)	58.3 ± 19.4	51.8 ± 18.0			
*P*_2_, Slope coefficient (mmHg^−1^)	Control (*n* = 10)	0.215 ± 0.242	0.196 ± 0.244	*p* = .217	*p* = .003	*p* = .107
	GSK1838705 (*n* = 12)	0.146 ± 0.031	0.088 ± 0.021			
*P*_3_, Midpoint pressure (mmHg)	Control (*n* = 10)	141.9 ± 17.0	139.5 ± 14.4	*p* = .461	*p* = .091	*p* = .301
	GSK1838705 (*n* = 12)	142.0 ± 10.5	132.3 ± 12.5			
*P*_4_, Minimum value (%)	Control (*n* = 10)	40.4 ± 24.9	42.7 ± 27.9	*p* = .651	*p* = .287	*p* = .115
	GSK1838705 (*n* = 12)	43.2 ± 18.1	31.7 ± 22.3			
*G*_max_, Maximum gain (%/mmHg)	Control (*n* = 10)	−2.38 ± 1.17	−2.39 ± 1.47	*p* = .077	*p* = .024	*p* = .021
	GSK1838705 (*n* = 12)	−2.17 ± 0.89	−1.14 ± 0.49*, ^†^			
*R* ^*2*^, Coefficient of determination	Control (*n* = 10)	0.964 ± 0.069	0.978 ± 0.040	*p* = .374	*p* = .600	*p* = .045
	GSK1838705 (*n* = 12)	0.989 ± 0.011	0.981 ± 0.014			
Peripheral arc						
*b*_0_, Intercept (mmHg)	Control (*n* = 10)	6.9 ± 33.6	10.4 ± 32.2	*p* = .990	*p* = .336	*p* = .760
	GSK1838705 (*n* = 12)	5.0 ± 32.5	11.9 ± 37.1			
*b*_1_, Slope (mmHg/%)	Control (*n* = 10)	1.354 ± 0.376	1.249 ± 0.398	*p* = .593	*p* = .823	*p* = .170
	GSK1838705 (*n* = 12)	1.345 ± 0.405	1.490 ± 0.801			
*R* ^*2*^, Coefficient of determination	Control (*n* = 10)	0.947 ± 0.076	0.954 ± 0.035	*p* = .639	*p* = .853	*p* = .509
	GSK1838705 (*n* = 12)	0.949 ± 0.031	0.937 ± 0.042			
Total reflex arc						
*P*_1_, Response range (mmHg)	Control (*n* = 11)	84.9 ± 34.7	72.2 ± 23.6	*p* = .315	*p* = .001	*p* = .977
	GSK1838705 (*n* = 13)	75.7 ± 17.0	62.9 ± 17.0			
*P*_2_, Slope coefficient (mmHg^−1^)	Control (*n* = 11)	0.182 ± 0.218	0.213 ± 0.267	*p* = .914	*p* = .435	*p* = .143
	GSK1838705 (*n* = 13)	0.240 ± 0.272	0.139 ± 0.049			
*P*_3_, Midpoint pressure (mmHg)	Control (*n* = 11)	137.7 ± 16.4	129.6 ± 13.4	*p* = .394	*p* = .004	*p* = .569
	GSK1838705 (*n* = 13)	135.5 ± 10.0	123.9 ± 13.2			
*P*_4_, Minimum value (mmHg)	Control (*n* = 11)	63.7 ± 29.0	70.1 ± 16.3	*p* = .826	*p* = .414	*p* = .300
	GSK1838705 (*n* = 13)	68.9 ± 16.6	68.1 ± 15.2			
*G*_max_, Maximum gain (mmHg/mmHg)	Control (*n* = 11)	−2.86 ± 1.84	−3.00 ± 2.56	*p* = .747	*p* = .224	*p* = .171
	GSK1838705 (*n* = 13)	−4.45 ± 5.71	−2.11 ± 0.77			
*R* ^*2*^, Coefficient of determination	Control (*n* = 11)	0.973 ± 0.073	0.974 ± 0.079	*p* = .377	*p* = .703	*p* = .767
	GSK1838705 (*n* = 13)	0.993 ± 0.006	0.993 ± 0.011			
HR control						
*P*_1_, Response range (bpm)	Control (*n* = 11)	43.0 ± 16.6	34.6 ± 12.0	*p* = .578	*p* = .031	*p* = .742
	GSK1838705 (*n* = 13)	39.0 ± 13.9	32.7 ± 16.1			
*P*_2_, Slope coefficient (mmHg^−1^)	Control (*n* = 11)	0.151 ± 0.168	0.180 ± 0.268	*p* = .386	*p* = .946	*p* = .127
	GSK1838705 (*n* = 13)	0.125 ± 0.035	0.099 ± 0.029			
*P*_3_, Midpoint pressure (mmHg)	Control (*n* = 11)	145.6 ± 19.7	141.8 ± 15.0	*p* = .325	*p* = .059	*p* = .441
	GSK1838705 (*n* = 13)	143.3 ± 8.5	134.5 ± 11.5			
*P*_4_, Minimum value (bpm)	Control (*n* = 11)	347.9 ± 28.3	357.0 ± 29.2	*p* = .158	*p* = .901	*p* = .017
	GSK1838705 (*n* = 13)	343.3 ± 17.9	333.3 ± 25.3^†^			
*G*_max_, Maximum gain (bpm/mmHg)	Control (*n* = 11)	−1.58 ± 2.16	−1.50 ± 2.27	*p* = .361	*p* < .001	*p* = .005
	GSK1838705 (*n* = 13)	−1.20 ± 0.46	−0.73 ± 0.28*			
*R* ^*2*^, Coefficient of determination	Control (*n* = 11)	0.992 ± 0.011	0.987 ± 0.011	*p* = .143	*p* = .215	*p* = .735
	GSK1838705 (*n* = 13)	0.986 ± 0.011	0.977 ± 0.030			

*Note*: The data were analyzed by a two‐way repeated measures ANOVA (solution‐by‐time) followed by Bonferroni's multiple comparison test.

* Significant difference from before (*p* < .05).

^†^ Significant difference from control (*p* < .05). Data are shown as the mean ± SD.

**FIGURE 5:**
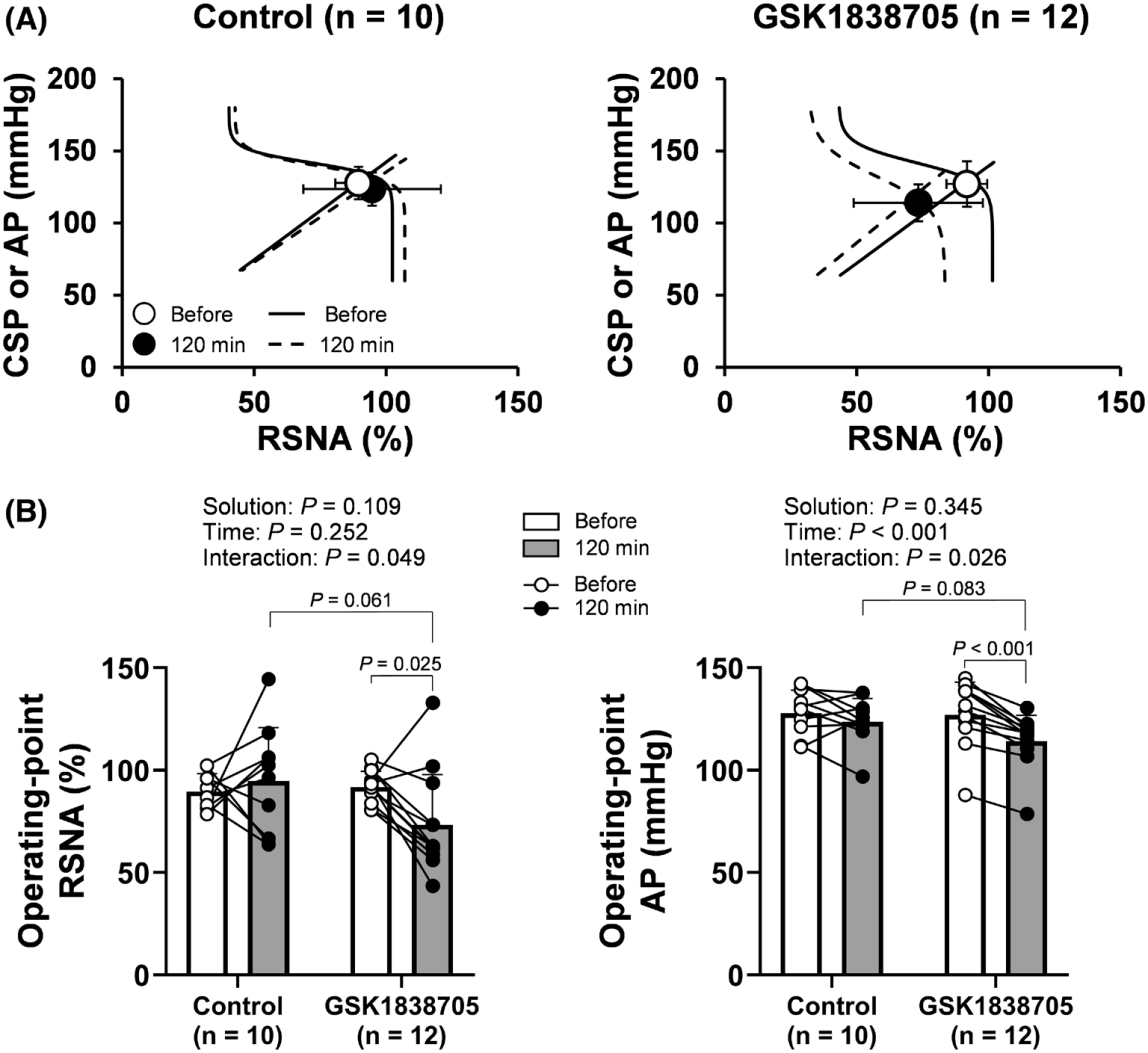
The baroreflex equilibrium diagram (A) and operating‐point arterial pressure (AP) and renal sympathetic nerve activity (RSNA) (B) before and 120 min after intracerebroventricular (ICV) injection of artificial cerebrospinal fluid (control) and insulin receptor antagonist (GSK1838705). The baroreflex equilibrium diagram was constructed from the fitted neural (sigmoid curve) and peripheral (linear regression line) arcs (A). In this diagram, the open and filled circles indicate operating‐point AP and RSNA before and 120 min after ICV injection of control and insulin receptor antagonist, respectively. The open (Before) and filled (120 min) circles represent individual data (B). The data were analyzed by a two‐way repeated measures ANOVA (solution‐by‐time) followed by Bonferroni's multiple comparison test. Data are shown as the mean ± SD.

Operating‐point RSNA and AP were determined from the baroreflex equilibrium diagram constructed from the fitted neural and peripheral arcs (Figure 5A). ICV injection of GSK1838705, but not the control solution, significantly decreased both operating‐point RSNA and AP (Figure 5B).

**FIGURE 6:**
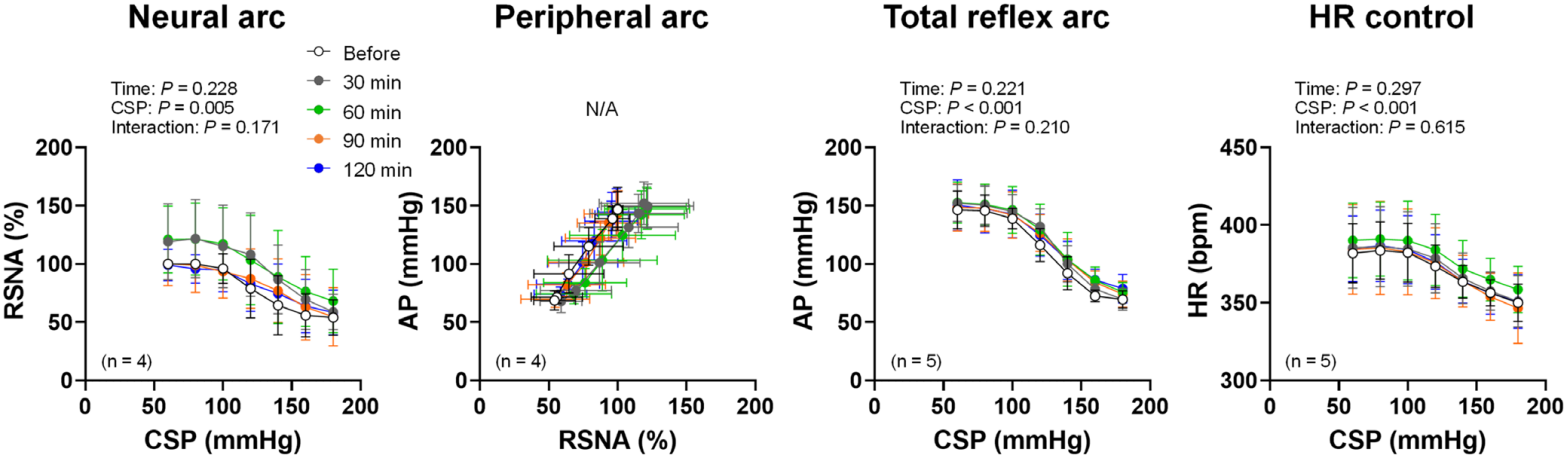
Open‐loop static characteristics of the neural arc, peripheral arc, total reflex arc, and heart rate (HR) control before and 30, 60, 90, and 120 min after intracerebroventricular injection of a 50% dimethyl sulfoxide solution. CSP, carotid sinus pressure; RSNA, renal sympathetic nerve activity; AP, arterial pressure. The open (Before) and filled (120 min) circles represent individual data. The data were analyzed by a two‐way repeated measures ANOVA (time‐by‐CSP). Since the x‐axis in the peripheral arc was RSNA, which varied between animals, a two‐way repeated measures ANOVA was not performed for the peripheral arc. Data are shown as the mean ± SD.

Figure 6 shows the trial‐averaged static characteristics of the baroreflex before and 30, 60, 90, and 120 min after ICV injection of a vehicle solution containing DMSO. We succeeded in measuring AP and HR in 5 rats and RSNA in 4 rats. The vehicle solution did not significantly change RSNA, AP, and HR responses to the stepwise CSP input. Moreover, none of the parameters of static characteristics of the neural arc, peripheral arc, total reflex arc, and HR arc, and operating‐point RSNA and AP were significantly altered after ICV injection of the vehicle solution (Table 2).

**TABLE 2:** Parameters of static characteristics of the neural arc, peripheral arc, total reflex arc, and heart rate (HR) control and operating‐point arterial pressure (AP) and renal sympathetic nerve activity (RSNA) before and 30, 60, 90, and 120 min after intracerebroventricular injection of 50% dimethyl sulfoxide solution.

	Before	30 min	60 min	90 min	120 min	*p* value
Neural arc (*n* = 4)						
*P*_1_, Response range (%)	50.2 ± 13.9	69.2 ± 22.1	60.3 ± 23.2	56.7 ± 8.5	41.4 ± 9.9	.262
*P*_2_, Slope coefficient (mmHg^−1^)	0.131 ± 0.070	0.068 ± 0.007	0.090 ± 0.031	0.069 ± 0.039	0.096 ± 0.036	.107
*P*_3_, Midpoint pressure (mmHg)	128.3 ± 20.2	138.4 ± 16.4	134.1 ± 21.1	145.1 ± 24.5	130.5 ± 19.6	.257
*P*_4_, Minimum value (%)	51.1 ± 14.4	52.7 ± 14.8	61.4 ± 31.2	44.4 ± 24.9	57.3 ± 20.8	.493
*G*_max_, Maximum gain (%/mmHg)	−1.53 ± 0.53	−1.16 ± 0.32	−1.32 ± 0.54	−0.96 ± 0.55	−1.04 ± 0.60	.359
*R* ^*2*^, Coefficient of determination	0.984 ± 0.016	0.986 ± 0.014	0.994 ± 0.004	0.982 ± 0.007	0.987 ± 0.012	.185
Peripheral arc (*n* = 4)						
*b*_0_, Intercept (mmHg)	−15.9 ± 32.6	−11.6 ± 8.3	−16.6 ± 35.4	−13.8 ± 28.7	−18.1 ± 29.0	.903
*b*_1_, Slope (mmHg/%)	1.611 ± 0.172	1.401 ± 0.336	1.374 ± 0.108	1.596 ± 0.264	1.656 ± 0.223	.265
*R* ^*2*^, Coefficient of determination	0.978 ± 0.007	0.963 ± 0.008	0.980 ± 0.008	0.985 ± 0.003	0.973 ± 0.008	.185
Total reflex arc (*n* = 5)						
*P*_1_, Response range (mmHg)	78.9 ± 15.9	84.2 ± 16.3	80.9 ± 15.1	82.4 ± 14.9	75.2 ± 19.9	.530
*P*_2_, Slope coefficient (mmHg^−1^)	0.109 ± 0.038	0.089 ± 0.019	0.090 ± 0.034	0.073 ± 0.026	0.081 ± 0.027	.304
*P*_3_, Midpoint pressure (mmHg)	128.8 ± 14.2	133.9 ± 9.5	132.5 ± 14.3	137.4 ± 13.6	131.4 ± 12.4	.451
*P*_4_, Minimum value (mmHg)	67.7 ± 7.5	67.4 ± 11.6	72.4 ± 11.4	67.3 ± 14.6	75.3 ± 15.6	.284
*G*_max_, Maximum gain (mmHg/mmHg)	−2.13 ± 0.71	−1.89 ± 0.59	−1.85 ± 0.83	−1.57 ± 0.73	−1.55 ± 0.71	.231
*R* ^*2*^, Coefficient of determination	0.999 ± 0.001	0.997 ± 0.005	0.997 ± 0.002	0.994 ± 0.007	0.997 ± 0.002	.385
HR control (*n* = 5)						
*P*_1_, Response range (bpm)	34.1 ± 17.4	35.8 ± 14.3	34.1 ± 11.6	44.2 ± 14.2	39.4 ± 12.7	.221
*P*_2_, Slope coefficient (mmHg^−1^)	0.315 ± 0.314	0.215 ± 0.233	0.177 ± 0.141	0.095 ± 0.043	0.096 ± 0.044	.475
*P*_3_, Midpoint pressure (mmHg)	132.8 ± 16.0	136.4 ± 16.4	139.3 ± 17.0	141.9 ± 18.8	136.6 ± 17.4	.411
*P*_4_, Minimum value (bpm)	349.1 ± 14.0	350.1 ± 17.2	357.0 ± 15.7	341.5 ± 23.0	346.9 ± 22.8	.126
*G*_max_, Maximum gain (bpm/mmHg)	−1.61 ± 1.13	−1.39 ± 0.71	−1.21 ± 0.49	−0.98 ± 0.46	−0.86 ± 0.31	.406
*R* ^*2*^, Coefficient of determination	0.963 ± 0.065	0.994 ± 0.007	0.993 ± 0.003	0.995 ± 0.005	0.994 ± 0.005	.938
Operating point (*n* = 4)						
Operating‐point RSNA (%)	83.2 ± 19.0	102.2 ± 32.0	103.1 ± 34.1	87.3 ± 25.6	85.5 ± 23.0	.493
Operating‐point AP (mmHg)	119.2 ± 7.7	123.8 ± 7.8	122.5 ± 12.4	121.5 ± 11.3	120.5 ± 12.0	.523

*Note*: The data were analyzed by a one‐way repeated measures ANOVA or Friedman test. Data are shown as the mean ± SD.

### 3.3 Baroreflex equilibrium diagram simulation under hypertensive and hypotensive stresses

**FIGURE 7:**
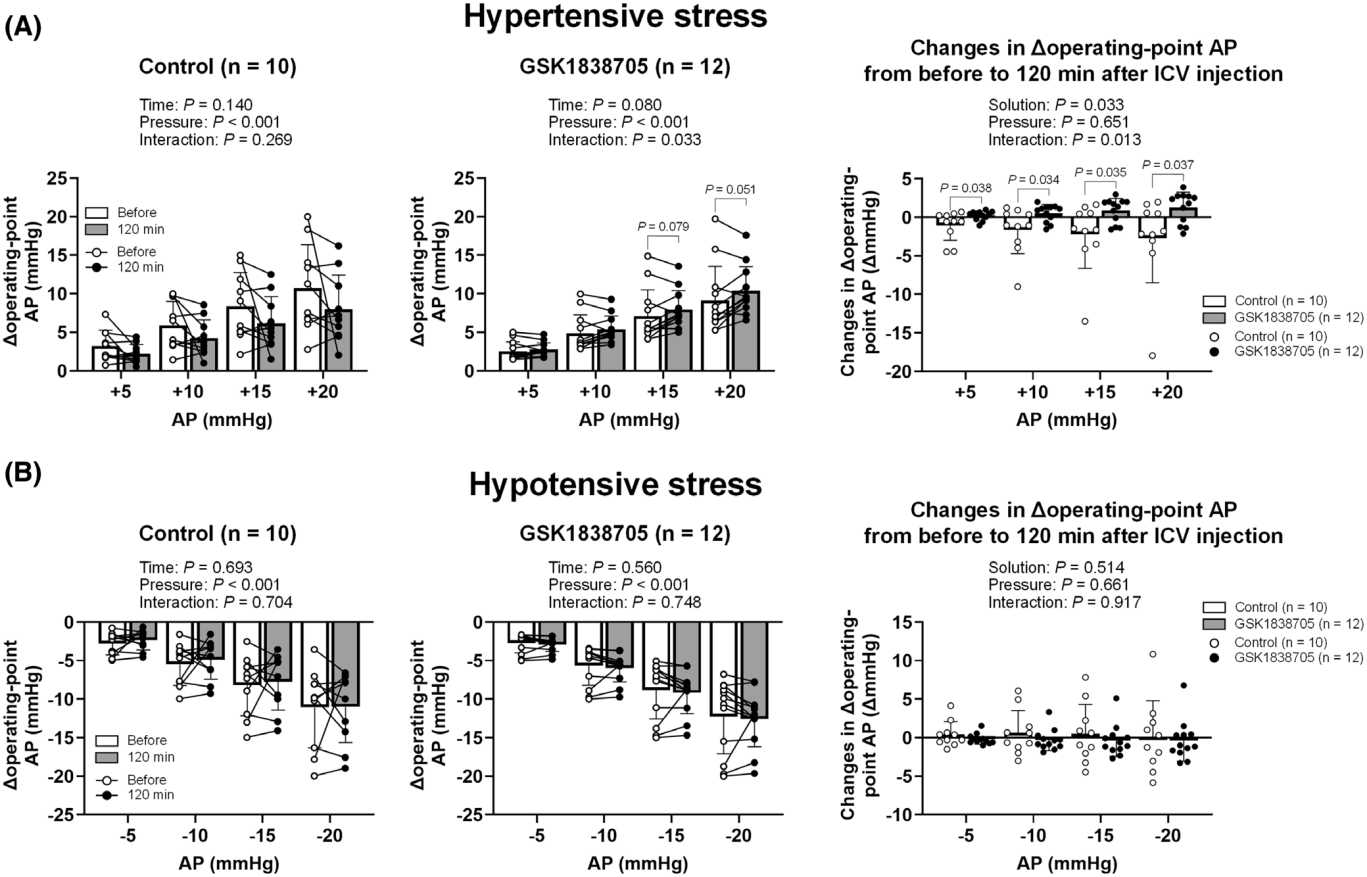
Baroreflex equilibrium diagram simulation under hypertensive (A) and hypotensive (B) stresses before and 120 min after intracerebroventricular injection of artificial cerebrospinal fluid (control) and insulin receptor antagonist (GSK1838705). The operating‐point arterial pressure (AP) under hypertensive/hypotensive stress was simulated by the intersection of the neural and peripheral arcs, whose intercept was shifted upward (+5, +10, +15, and +20 mmHg [hypertensive stress]) and downward (−5, −10, −15, and −20 mmHg [hypotensive stress]) in the baroreflex equilibrium diagram. Δoperating‐point AP; the difference in operating‐point AP from before to after hypertensive and hypotensive stresses. The data were analyzed by a two‐way repeated measures ANOVA (time‐by‐pressure or solution‐by‐pressure) followed by Bonferroni's multiple comparison test. Data are shown as the mean ± SD.

Figure 7A shows the simulation of hypertensive stress before and 120 min after ICV injection of control and IR antagonist solutions. Although Δoperating‐point AP was not significantly changed by ICV injection of control solution under hypertensive stress, there was a significant interaction in the GSK1838705 trial. Furthermore, the changes in Δoperating‐point AP from before to 120 min after ICV injection in the GSK1838705 trial were significantly higher than those in the control trial under all levels of hypertensive stress (+5, +10, +15, and +20 mmHg) (Figure 7A). Δoperating‐point AP was not significantly altered by ICV injection of control and GSK1838705 solutions under any levels of hypotensive stress (−5, −10, −15, and −20 mmHg) (Figure 7B).

## 4 DISCUSSION

The major findings from this investigation are as follows: 1) IR and c‐Fos activated via baroreflex stimulation by stepwise CSP input were observed to co‐localize in the NTS, CVLM, and RVLM; 2) ICV injection of an IR antagonist acutely decreased RSNA, AP, and HR during stepwise CSP input; 3) blockade of IR in the brain acutely impaired neural arc function but not peripheral arc function; 4) the operating‐point RSNA and AP were acutely decreased by IR blockade in the brain under open‐loop conditions; and 5) ICV injection of an IR antagonist increased the operating‐point AP rise by hypertensive stress using numerical simulation. Importantly, the effects of the IR antagonist were independent of circulating insulin and glucose levels. To date, brain insulin levels have been suggested to modulate arterial baroreflex function. The present findings further suggest that not only the availability of insulin but also brain IR signaling is important for arterial baroreflex control of AP.

It is well known that the signal from carotid sinus baroreceptors projects via sensory afferents to the NTS followed by CVLM and then RVLM in the sympathetic baroreflex function.^17^, ^18^ As such, we examined whether repetitive baroreflex stimulation activates c‐Fos in central neurons, and whether c‐Fos co‐localizes with IR. Activation of the arterial baroreflex has been reported to increase c‐Fos in the NTS, CVLM, and RVLM.^33^, ^34^, ^35^, ^36^, ^37^ Consistent with these findings, we observed that c‐Fos‐positive neurons in the NTS, CVLM, and RVLM were increased by 120‐min stepwise CSP input stimulation under open‐loop conditions. To the best of our knowledge, this is the first study showing that the NTS, CVLM, and RVLM are populated with IR‐positive neurons co‐expressing c‐Fos that are activated by baroreflex stimulation.

### 4.1 Possible mechanism of impairing arterial baroreflex function by brain IR blockade

It has long been appreciated that acute ICV insulin infusion increases SNA.^2^, ^3^, ^4^, ^5^ Rahmouni et al.^4^ demonstrated that ICV administration of a high dose of insulin (500 mU) increases RSNA in rats. We found that ICV injection of an IR antagonist significantly reduced RSNA during stepwise CSP input. Furthermore, the operating‐point RSNA was decreased by the IR antagonist. It is well known that changes in the circulating levels of glucose and insulin alter SNA.^38^, ^39^, ^40^ We found that ICV injection of GSK1838705 did not significantly change blood glucose and plasma insulin levels in the present study. Thus, it is plausible that the decrease in RSNA is induced by impairment of IR signaling in the brain, independent of circulating glucose and insulin levels.

The most important finding of this study is that the maximum gain of the neural arc was decreased by ICV injection of an IR antagonist, indicating that the neural arc function was impaired by IR blockade in the brain. In contrast, the slope and intercept of the regression line in the peripheral arc were not altered by the IR antagonist GSK1838705. The neural arc determines the SNA response to baroreceptor pressure input, while the peripheral arc determines AP as a result of cardiovascular responses to SNA input.^16^ Thus, these results suggest that IR signaling in the brain is involved in arterial baroreflex function via central nervous system mechanisms without affecting peripheral SNA‐induced vasoconstrictive function. It is noted that the operating‐point RSNA was significantly decreased by ICV injection of the IR antagonist. This suggests that the blockade of IR in the brain reduces baseline RSNA. Thus, it is challenging to exclude the possibility that the reduction of baseline RSNA per se would attenuate the neural arc gain. However, this is beyond the scope of the present investigation, and further research is warranted.

We recently found that IRs are highly expressed in the NTS, and that microinjection of an IR antagonist (GSK1838705) into the NTS acutely increases the pressor response to electrically induced muscle contractions in baroreceptor‐intact rats but not in denervated rats.^22^ The results suggest that acute blockade of IR signaling in the NTS increases the exercise pressor reflex through interactions with baroreflex neurons in the NTS. Sensory information from the carotid sinus nerve projects to the NTS and further to the CVLM within the medulla oblongata. These neurons then project to the RVLM.^17^, ^18^ Critical neurotransmission from the CVLM to the RVLM is mediated through the inhibitory amino acid GABA, which inhibits sympathoexcitatory neurons in the RVLM, resulting in a decrease in SNA and AP.^17^, ^18^ The binding of insulin to IR activates phosphoinositide 3‐kinase, which converts phosphorylated phosphatidylinositol 4,5‐bisphosphate into (3,4,5)‐trisphosphate (PIP3) through phosphorylation.^41^, ^42^ PIP3 is known to activate ATP‐dependent potassium channels, resulting in hyperpolarization of neurons and decreased neuronal firing rate.^43^ Furthermore, it has been demonstrated that the microinjection of insulin into the NTS decreases the spontaneous discharge of baroreflex‐sensitive NTS neurons in anesthetized rats.^44^ Therefore, although speculative in nature, ICV injection of an IR antagonist may inhibit IR signaling‐induced decreases in NTS neuron activation by reducing hyperpolarization, which in turn reduces the activation of sympathoexcitatory neurons in the RVLM, resulting in the inhibition of the reflex response to changes in CSP. Furthermore, astrocytes in the NTS have been suggested to play a role in the arterial baroreflex.^45^, ^46^ NTS astrocytes might be involved in the brain IR blockade‐induced impairment of arterial baroreflex function. Alternatively, as Cassaglia et al.^47^ reported that the arcuate nucleus is the primary site of action where insulin enhances baroreflex function, it is therefore possible that blockade of IR signaling in the arcuate nucleus could also lead to impairment of arterial baroreflex function. In contrast, it has been reported that the nanoinjection of an IR antagonist into the arcuate nucleus does not significantly decrease lumbar SNA, AP, and HR in anesthetized healthy rats.^48^ Thus, it is unlikely that the IR antagonist‐induced arterial baroreflex dysfunction can be fully explained by its action in the arcuate nucleus. Of note, however, our study did not identify the site of action of the IR antagonist in the brain. Future studies should investigate this point.

Blockade of IR in the brain decreased both AP during CSP input and operating‐point AP, which is consistent with results of the neural arc and operating‐point RSNA. Since previous studies examined the effects of brain insulin on the baroreflex under closed‐loop conditions, the arterial baroreflex control of AP has not been investigated. To the best of our knowledge, therefore, our study is the first to demonstrate that brain IR signaling has a crucial role in AP regulation via arterial baroreflex control of SNA. The operating point under open‐loop conditions is determined from the intersection between the neural and peripheral arcs on the baroreflex equilibrium diagram.^16^, ^31^ The present study showed that ICV injection of IR antagonist altered the neural arc by attenuating the maximum gain while the peripheral arc did not change. Therefore, the brain IR blockade‐induced reduction in AP may occur by impairing the neural arc function in arterial baroreflex control of SNA.

IR antagonist injection in the brain impairs HR control by reducing the maximum gain. Since bilateral vagal and aortic depressor nerves were sectioned in this study, the changes in HR would mainly result from its response to CSP via SNA. Although we did not measure cardiac SNA, a previous study reported that the open‐loop static characteristics of baroreflex control of cardiac SNA parallel those of RSNA.^49^ Hence, IR blockade may impair arterial baroreflex control of HR via cardiac SNA. Furthermore, this is consistent with an earlier study, which found that baroreflex gain of HR was decreased in pregnant rabbits with a decrease in CSF insulin.^9^ Taken together, our findings suggest that arterial baroreflex control of HR is impaired by decreasing brain IR signaling.

RSNA during stepwise CSP input was significantly decreased at 120 min after ICV injection of the IR antagonist, while AP and HR were significantly reduced from 30 to 60 min after the injection. It has been shown that the activation of RSNA is slowly increased by ICV injection of insulin compared to lumbar SNA.^4^ In addition, Pricher et al.^8^ demonstrated that the gain of baroreflex control of HR and lumbar SNA was improved within 60 min after brain insulin infusion. Therefore, it is plausible, albeit speculative, that decreased lumbar and cardiac SNA might induce attenuation of AP and HR before a remarkable decrease in RSNA. That being said, 120 min after IR antagonist injection, the changes in the total reflex arc were similar to those of the neural arc. Additionally, both operating‐point RSNA and AP were decreased. Therefore, there is no doubt that the decrease in AP is at least partially attributable to the attenuation of RSNA.

### 4.2 Clinical implications

Our findings suggest that IR signaling in the brain plays a crucial role in AP regulation via modulation of arterial baroreflex function. Arterial baroreflex dysfunction has been well documented to induce AP lability.^6^ AP lability is often observed in patients with diabetes mellitus (DM), leading to orthostatic hypotension and exercise intolerance.^15^ Moreover, evidence suggests that high short‐term AP variability increases the risk of cardiovascular disease in DM patients.^50^, ^51^ Circulating insulin is transported into the brain across the blood–brain barrier.^52^ Hyperinsulinemia and peripheral insulin resistance, particularly in the early stage of type 2 DM, have been reported to induce a deficit in insulin transport across the blood–brain barrier.^53^, ^54^, ^55^ Increasing evidence suggests that reduced brain insulin levels impair arterial baroreflex function.^9^, ^10^ Notably, earlier studies have demonstrated that type 1 and type 2 DM animals have decreased brain IR signaling, suggesting that DM induces an impaired IR signaling pathway in the brain.^13^, ^14^ Taken together with the findings of the present study, the decrease not only in brain insulin availability but also in IR signaling in the medullary cardiovascular centers may, at least in part, induce AP lability in this disease.

In this study, the numerical simulation using the baroreflex equilibrium diagram revealed that brain IR antagonist raised the operating‐point AP in response to hypertensive stress. This suggests that brain IR blockade may reduce the responsiveness of sympathetic baroreflex regulation against external perturbation, especially in the high AP range. The exaggerated AP response to exercise has often been observed in patients^56^, ^57^, ^58^ as well as animals^59^, ^60^, ^61^, ^62^, ^63^ with DM. Therefore, IR signaling dysfunction in the brain seen in DM might partially explain the AP lability in the high AP range. Since we studied only normal healthy rats in the present investigation, future investigations should track the causal relationship between brain IR signaling dysfunction and baroreflex failure using animals and patients with DM.

### 4.3 Limitations

We also acknowledge several limitations in the present study. First, the acute effect of IR blockade in the brain on the arterial baroreflex was investigated in anesthetized rats. Thus, the present results may not be directly applicable to conscious individuals. Second, as discussed above, because we performed ICV injection for delivering IR antagonist into the brain, the site of action in the brain was not identified. Third, only male rats were used in this study. Because the menstrual/estrous cycle has been suggested to influence baroreflex function,^64^, ^65^ it is unknown whether the interpretation of our results applies to females. Further studies are required to elucidate these points. Fourth, the stepwise CSP input was repeated for 120 min in the present study. Prolonged baroreflex activation has been reported to acutely decrease AP levels.^66^ Although open‐loop baroreflex function and operating‐point AP and RSNA did not significantly change over 120 min in the control trial, the potential impact of repeated CSP input on arterial baroreflex function, as well as the operating‐point AP or RSNA, cannot be fully excluded. Lastly, GSK1838705 was diluted in aCSF containing 50% DMSO. To investigate the effect of DMSO, aCSF with a 50% DMSO solution was administered intraventricularly. As a result, the significant effects seen with ICV injection of GSK1838705 were not observed. Additionally, given the total CSF volume in adult rats,^67^ the concentration of DMSO after dilution in brain CSF would be expected to be less than about 0.5%. As such, it is unlikely that DMSO caused transient impairments in arterial baroreflex function.

## 5 CONCLUSION

The data demonstrate that the blockade of IR in the brain acutely alters arterial baroreflex function and decreases operating‐point RSNA and AP by impairing the neural arc function under open‐loop conditions. These results suggest that IR signaling in the brain plays an important role in AP regulation via the neural arc function of arterial baroreflex.

## AUTHOR CONTRIBUTIONS

AH and MM conceived and designed experiments. AH performed experiments. AH and MM analyzed data. AH, TK, NH, AF, JAE, HKK, GAI, SAS, WV, and MM interpreted the results of experiments. AH and MM prepared figures. AH and MM drafted the manuscript or revised it critically for important intellectual content. AH, TK, NH, AF, JAE, HKK, GAI, SAS, WV, and MM have read and approved the final version of this manuscript.

## DISCLOSURES

TK received a consulting fee from NTT Research, Inc.

## Data Availability

The data supporting the present findings are available from the corresponding author upon reasonable request.

## References

[1] Saltiel AR , Kahn CR . Insulin signalling and the regulation of glucose and lipid metabolism. Nature. 2001;414:799‐806. 11742412 10.1038/414799a

[2] Muntzel MS , Morgan DA , Mark AL , Johnson AK . Intracerebroventricular insulin produces nonuniform regional increases in sympathetic nerve activity. Am J Physiol Regul Integr Comp Physiol. 1994;267:R1350‐R1355. 10.1152/ajpregu.1994.267.5.R1350 7977864

[3] Muntzel M , Beltz T , Mark AL , Johnson AK . Anteroventral third ventricle lesions abolish lumbar sympathetic responses to insulin. Hypertension. 1994;23:1059‐1062. 8206594 10.1161/01.hyp.23.6.1059

[4] Rahmouni K , Morgan DA , Morgan GM , et al. Hypothalamic PI3K and MAPK differentially mediate regional sympathetic activation to insulin. J Clin Invest. 2004;114:652‐658. 15343383 10.1172/JCI21737 PMC514588

[5] Ward KR , Bardgett JF , Wolfgang L , Stocker SD . Sympathetic response to insulin is mediated by melanocortin 3/4 receptors in the hypothalamic paraventricular nucleus. Hypertension. 2011;57:435‐441. 21263116 10.1161/HYPERTENSIONAHA.110.160671 PMC3580160

[6] Lanfranchi PA , Somers VK . Arterial baroreflex function and cardiovascular variability: interactions and implications. Am J Physiol Regul Integr Comp Physiol. 2002;283:R815‐R826. 12228049 10.1152/ajpregu.00051.2002

[7] Stevens SL , Wood S , Koshiaris C , et al. Blood pressure variability and cardiovascular disease: systematic review and meta‐analysis. BMJ. 2016;354:14‐16. 10.1136/bmj.i4098 PMC4979357 27511067

[8] Pricher MP , Freeman KL , Brooks VL . Insulin in the brain increases gain of baroreflex control of heart rate and lumbar sympathetic nerve activity. Hypertension. 2008;51:514‐520. 18158342 10.1161/HYPERTENSIONAHA.107.102608

[9] Daubert DL , Chung MY , Brooks VL . Insulin resistance and impaired baroreflex gain during pregnancy. Am J Physiol Regul Integr Comp Physiol. 2007;292:2188‐2195. 10.1152/ajpregu.00614.2006 17303682

[10] Azar AS , Brooks VL . Impaired baroreflex gain during pregnancy in conscious rats: role of brain insulin. Hypertension. 2011;57:283‐288. 21149828 10.1161/HYPERTENSIONAHA.110.162354 PMC3024461

[11] Pirola L , Johnston AM , Van Obberghen E . Modulation of insulin action. Diabetologia. 2004;47:170‐184. 14722654 10.1007/s00125-003-1313-3

[12] Plum L , Schubert M , Brüning JC . The role of insulin receptor signaling in the brain. Trends Endocrinol Metab. 2005;16:59‐65. 15734146 10.1016/j.tem.2005.01.008

[13] Liu Y , Liu F , Grundke‐Iqbal I , Iqbal K , Gong CX . Deficient brain insulin signalling pathway in Alzheimer's disease and diabetes. J Pathol. 2011;225:54‐62. 21598254 10.1002/path.2912 PMC4484598

[14] Li ZG , Zhang W , Sima AA . Alzheimer‐like changes in rat models of spontaneous diabetes. Diabetes. 2007;56:2650. 10.2337/db07-0171 17456849

[15] Vinik AI , Ziegler D . Diabetic cardiovascular autonomic neuropathy. Circulation. 2007;115:387‐397. 17242296 10.1161/CIRCULATIONAHA.106.634949

[16] Kawada T , Sugimachi M . Open‐loop static and dynamic characteristics of the arterial baroreflex system in rabbits and rats. J Physiol Sci. 2016;66:15‐41. 26541155 10.1007/s12576-015-0412-5 PMC4742515

[17] Pilowsky PM , Goodchild AK . Baroreceptor reflex pathways and neurotransmitters: 10 years on. J Hypertens. 2002;20:1675‐1688. 12195099 10.1097/00004872-200209000-00002

[18] Wehrwein EA , Joyner MJ . Regulation of blood pressure by the arterial baroreflex and autonomic nervous system. Handb Clin Neurol. 2013;117:89‐102. 24095118 10.1016/B978-0-444-53491-0.00008-0

[19] Werther GA , Hogg A , Oldfield BJ , et al. Localization and characterization of insulin receptors in rat brain and pituitary gland using in vitro autoradiography and computerized densitometry. Endocrinology. 1987;121:1562‐1570. 3653038 10.1210/endo-121-4-1562

[20] Sharp FR , Sagar SM , Hicks K , Lowenstein D , Hisanaga K . c‐fos mRNA, Fos, and Fos‐related antigen induction by hypertonic saline and stress. J Neurosci. 1991;11:2321‐2331. 1908006 10.1523/JNEUROSCI.11-08-02321.1991 PMC6575523

[21] Herrera DG , Robertson HA . Activation of c‐fos in the brain. Prog Neurobiol. 1996;50:83‐107. 8971979 10.1016/s0301-0082(96)00021-4

[22] Estrada JA , Hotta N , Kim K , et al. Blockade of endogenous insulin receptor signaling in the nucleus tractus solitarius potentiates exercise pressor reflex function in healthy male rats. FASEB J. 2023;37:e23141. 37566482 10.1096/fj.202300879RR PMC10430879

[23] Murphy MN , Mizuno M , Downey RM , Squiers JJ , Squiers KE , Smith SA . Neuronal nitric oxide synthase expression is lower in areas of the nucleus tractus solitarius excited by skeletal muscle reflexes in hypertensive rats. Am J Physiol Heart Circ Physiol. 2013;304:1547‐1557. 10.1152/ajpheart.00235.2012 PMC3680727 23564306

[24] Paxinos G , Watson C . The Rat Brain in Stereotaxic Coordinates: Compact. 6th ed. Elsevier; 2009.

[25] Kawada T , Li M , Kamiya A , et al. Open‐loop dynamic and static characteristics of the carotid sinus baroreflex in rats with chronic heart failure after myocardial infarction. J Physiol Sci. 2010;60:283‐298. 20514557 10.1007/s12576-010-0096-9 PMC10717991

[26] Kawada T , Shimizu S , Li M , et al. Contrasting effects of moderate vagal stimulation on heart rate and carotid sinus baroreflex‐mediated sympathetic arterial pressure regulation in rats. Life Sci. 2011;89:498‐503. 21855552 10.1016/j.lfs.2011.07.026

[27] Sato T , Kawada T , Miyano H , et al. New simple methods for isolating baroreceptor regions of carotid sinus and aortic depressor nerves in rats. Am J Physiol Heart Circ Physiol. 1999;276:H326‐H332. 10.1152/ajpheart.1999.276.1.H326 9887047

[28] Kawada T , Li M , Zheng C , et al. Chronic vagal nerve stimulation improves baroreflex neural arc function in heart failure rats. J Appl Physiol. 2014;116:1308‐1314. 24674859 10.1152/japplphysiol.00140.2014

[29] Kawada T , Shimizu S , Yamamoto H , Miyamoto T , Shishido T , Sugimachi M . Peripheral versus central effect of intravenous moxonidine on rat carotid sinus baroreflex‐mediated sympathetic arterial pressure regulation. Life Sci. 2017;190:103‐109. 28964815 10.1016/j.lfs.2017.09.038

[30] Kent BB , Drane JW , Blumenstein B , Manning JW . A mathematical model to assess changes in the baroreceptor reflex. Cardiology. 1972;57:295‐310. 4651782 10.1159/000169528

[31] Sato T , Kawada T , Inagaki M , et al. New analytic framework for understanding sympathetic baroreflex control of arterial pressure. Am J Physiol Heart Circ Physiol. 1999;276:2251‐2261. 10.1152/ajpheart.1999.276.6.H2251 10362709

[32] Kamada K , Saku K , Tohyama T , et al. Diabetes mellitus attenuates the pressure response against hypotensive stress by impairing the sympathetic regulation of the baroreflex afferent arc. Am J Physiol Heart Circ Physiol. 2019;316:H35‐H44. 30339460 10.1152/ajpheart.00515.2018

[33] Wei S , Lei M , Tong M , Ding J , Han Q , Xiao M . Acute baroreceptor unloading evokes Fos expression in anesthetized rat brain. Brain Res Bull. 2008;76:63‐69. 18395612 10.1016/j.brainresbull.2007.12.003

[34] Minson JB , Llewellyn‐Smith IJ , Arnolda LF , Pilowsky PM , Chalmers JP . C‐fos expression in central neurons mediating the arterial baroreceptor reflex. Clin Exp Hypertens. 1997;19:631‐643. 9247744 10.3109/10641969709083175

[35] Chan JYH , Chen WC , Lee HY , Chan SHH . Elevated Fos expression in the nucleus tractus solitarii is associated with reduced baroreflex response in spontaneously hypertensive rats. Hypertension. 1998;32:939‐944. 9822457 10.1161/01.hyp.32.5.939

[36] Li YW , Dampney RAL . Expression of fos‐like protein in brain following sustained hypertension and hypotension in conscious rabbits. Neuroscience. 1994;61:613‐634. 7969933 10.1016/0306-4522(94)90439-1

[37] Dean C , Seagard JL . Expression of c‐fos protein in the nucleus tractus solitarius in response to physiological activation of carotid baroreceptors. Neuroscience. 1995;69:249‐257. 8637623 10.1016/0306-4522(95)00217-7

[38] Morgan DA , Balon TW , Ginsberg BH , Mark AL . Nonuniform regional sympathetic nerve responses to hyperinsulinemia in rats. Am J Phys. 1993;264:R423‐R427. 10.1152/ajpregu.1993.264.2.R423 8447499

[39] Rowe JW , Young JB , Minaker KL , Stevens AL , Pallotta J , Landsberg L . Effect of insulin and glucose infusions on sympathetic nervous system activity in normal man. Diabetes. 1981;30:219‐225. 7009270 10.2337/diab.30.3.219

[40] Lu H , Duanmu Z , Scislo T , Dunbar JC . The co‐existence of insulin‐mediated decreased mean arterial pressure and increased sympathetic nerve activity is not mediated by the baroreceptor reflex and differentially by hypoglycemia. Clin Exp Hypertens. 1998;20:165‐183. 9533612 10.3109/10641969809053213

[41] Boucher J , Kleinridders A , Kahn CR . Insulin receptor signaling in normal and insulin‐resistant states. Cold Spring Harb Perspect Biol. 2014;6:a009191. 24384568 10.1101/cshperspect.a009191 PMC3941218

[42] Muniyappa R , Montagnani M , Koh KK , Quon MJ . Cardiovascular actions of insulin. Endocr Rev. 2007;28:463‐491. 17525361 10.1210/er.2007-0006

[43] Plum L , Ma X , Hampel B , et al. Enhanced PIP3 signaling in POMC neurons causes KATP channel activation and leads to diet‐sensitive obesity. J Clin Invest. 2006;116:1886‐1901. 16794735 10.1172/JCI27123 PMC1481658

[44] Ruggeri P , Molinari C , Brunori A , et al. The direct effect of insulin on barosensitive neurones in the nucleus tractus solitarii of rats. Neuroreport. 2001;12:3719‐3722. 11726781 10.1097/00001756-200112040-00023

[45] Lin LH , Moore SA , Jones SY , McGlashon J , Talman WT . Astrocytes in the rat nucleus tractus solitarii are critical for cardiovascular reflex control. J Neurosci. 2013;33:18608‐18617. 24259582 10.1523/JNEUROSCI.3257-13.2013 PMC3834061

[46] Mastitskaya S , Turovsky E , Marina N , et al. Astrocytes modulate baroreflex sensitivity at the level of the nucleus of the solitary tract. J Neurosci. 2020;40:3052‐3062. 32132265 10.1523/JNEUROSCI.1438-19.2020 PMC7141885

[47] Cassaglia PA , Hermes SM , Aicher SA , Brooks VL . Insulin acts in the arcuate nucleus to increase lumbar sympathetic nerve activity and baroreflex function in rats. J Physiol. 2011;589:1643‐1662. 21300750 10.1113/jphysiol.2011.205575 PMC3099021

[48] Shi Z , Zhao D , Cassaglia PA , Brooks VL . Sites and sources of sympathoexcitation in obese male rats: role of brain insulin. Am J Physiol Regul Integr Comp Physiol. 2020;318:R634‐R648. 31967846 10.1152/ajpregu.00317.2019 PMC7099464

[49] Kamiya A , Kawada T , Yamamoto K , et al. Dynamic and static baroreflex control of muscle sympathetic nerve activity (SNA) parallels that of renal and cardiac SNA during physiological change in pressure. Am J Physiol Heart Circ Physiol. 2005;289:2641‐2648. 10.1152/ajpheart.00642.2005 16055514

[50] Chiriacò M , Pateras K , Virdis A , et al. Association between blood pressure variability, cardiovascular disease and mortality in type 2 diabetes: a systematic review and meta‐analysis. Diabetes Obes Metab. 2019;21:2587‐2598. 31282073 10.1111/dom.13828

[51] Parati G , Ochoa JE , Salvi P , Lombardi C , Bilo G . Prognostic value of blood pressure variability and average blood pressure levels in patients with hypertension and diabetes. Diabetes Care. 2013;36:S312‐S324. 23882065 10.2337/dcS13-2043 PMC3920798

[52] Gray SM , Barrett EJ . Insulin transport into the brain. Am J Physiol Cell Physiol. 2018;315:C125‐C136. 29847142 10.1152/ajpcell.00240.2017 PMC6139500

[53] Kaiyala KJ , Prigeon RL , Kahn SE , Woods SC , Schwartz MW . Obesity induced by a high‐fat diet is associated with reduced brain insulin transport in dogs. Diabetes. 2000;49:1525‐1533. 10969837 10.2337/diabetes.49.9.1525

[54] Heni M , Schöpfer P , Peter A , et al. Evidence for altered transport of insulin across the blood‐brain barrier in insulin‐resistant humans. Acta Diabetol. 2014;51:679‐681. 24370925 10.1007/s00592-013-0546-y

[55] Kern W , Benedict C , Schultes B , et al. Low cerebrospinal fluid insulin levels in obese humans. Diabetologia. 2006;49:2790‐2792. 16951936 10.1007/s00125-006-0409-y

[56] Holwerda SW , Restaino RM , Manrique C , Lastra G , Fisher JP , Fadel PJ . Augmented pressor and sympathetic responses to skeletal muscle metaboreflex activation in type 2 diabetes patients. Am J Physiol Heart Circ Physiol. 2016;310:H300‐H309. 26566729 10.1152/ajpheart.00636.2015 PMC5504388

[57] Matteucci E , Rosada J , Pinelli M , Giusti C , Giampietro O . Systolic blood pressure response to exercise in type 1 diabetes families compared with healthy control individuals. J Hypertens. 2006;24:1745‐1751. 16915023 10.1097/01.hjh.0000242398.60838.5d

[58] Scott JA , Coombes JS , Prins JB , Leano RL , Marwick TH , Sharman JE . Patients with type 2 diabetes have exaggerated brachial and central exercise blood pressure: relation to left ventricular relative wall thickness. Am J Hypertens. 2008;21:715‐721. 18437126 10.1038/ajh.2008.166

[59] Kim HK , Hotta N , Ishizawa R , et al. Exaggerated pressor and sympathetic responses to stimulation of the mesencephalic locomotor region and exercise pressor reflex in type 2 diabetic rats. Am J Physiol Regul Integr Comp Physiol. 2019;317:R270‐R279. 31091155 10.1152/ajpregu.00061.2019

[60] Ishizawa R , Kim HK , Hotta N , et al. Skeletal muscle reflex–induced sympathetic dysregulation and sensitization of muscle afferents in type 1 diabetic rats. Hypertension. 2020;75:1072‐1081. 32063060 10.1161/HYPERTENSIONAHA.119.14118 PMC7117783

[61] Ishizawa R , Kim HK , Hotta N , et al. TRPV1 (transient receptor potential vanilloid 1) sensitization of skeletal muscle afferents in type 2 diabetic rats with hyperglycemia. Hypertension. 2021;1:1360‐1371. 10.1161/HYPERTENSIONAHA.120.15672 PMC8109832 33641357

[62] Mizuno M , Hotta N , Ishizawa R , et al. The impact of insulin resistance on cardiovascular control during exercise in diabetes. Exerc Sport Sci Rev. 2021;49:157‐167. 33965976 10.1249/JES.0000000000000259 PMC8195845

[63] Estrada JA , Ishizawa R , Kim HK , et al. Intracerebroventricular insulin injection acutely normalizes the augmented exercise pressor reflex in male rats with type 2 diabetes mellitus. J Physiol. 2024. doi:10.1113/JP286715 PMC12400197 39165238

[64] Minson CT , Halliwill JR , Young TM , Joyner MJ . Influence of the menstrual cycle on sympathetic activity, baroreflex sensitivity, and vascular transduction in young women. Circulation. 2000;101:862‐868. 10694525 10.1161/01.cir.101.8.862

[65] Goldman RK , Azar AS , Mulvaney JM , Hinojosa‐Laborde C , Haywood JR , Brooks VL . Baroreflex sensitivity varies during the rat estrous cycle: role of gonadal steroids. Am J Physiol Regul Integr Comp Physiol. 2009;296:1419‐1426. 10.1152/ajpregu.91030.2008 PMC2689823 19261912

[66] Lohmeier TE , Irwin ED , Rossing MA , Serdar DJ , Kieval RS . Prolonged activation of the baroreflex produces sustained hypotension. Hypertension. 2004;43:306‐311. 14707159 10.1161/01.HYP.0000111837.73693.9b

[67] Barten DM , Cadelina GW , Weed MR . Dosing, collection, and quality control issues in cerebrospinal fluid research using animal models. Handb Clin Neurol. 2017;146:3‐20. 29110779 10.1016/B978-0-12-804279-3.00004-6

